# Low socioeconomic status, insulin resistance, asthma, and depression: A syndemic framework in Brazilian adolescents

**DOI:** 10.1111/pai.70290

**Published:** 2026-02-22

**Authors:** Aline Sousa Falcão, Lorena Lúcia Costa Ladeira, Susilena Arouche Costa, Rosângela Fernandes Lucena Batista, Claudia Maria Coelho Alves, Erika Bárbara Abreu Fonseca Thomaz, Cecília Claudia Costa Ribeiro

**Affiliations:** ^1^ Graduate Program in Public Health Federal University of Maranhão São Luís Brazil; ^2^ Graduate Program in Dentistry Federal University of Maranhão São Luís Brazil

**Keywords:** adolescents, asthma, depression, insulin resistance, Syndemia

## Abstract

**Background:**

Adolescence is a critical developmental stage during which environmental, metabolic, and psychosocial factors co‐occur and may interact, influencing health and creating a syndemic context that remains insufficiently explored.

**Objective:**

We analyzed a syndemic model, considering socioeconomic determinants and metabolic risk associated with asthma and depression in adolescents.

**Methods:**

This population‐based study (*n* = 2515) utilized data from the RPS Brazilian Consortium Cohorts at follow‐up of 18–19 years in São Luís, Brazil. A theoretical model analyzed the association among socioeconomic determinants, metabolic risk, asthma, and depression using Structural Equation Modeling (SEM). *Low Socioeconomic Status* was a distal determinant. The metabolic risk included the TG/HDL ratio, as an insulin resistance marker, as well as obesity. *Asthma* was a latent variable based on wheezing, asthma history, medical diagnosis, and frequency of wheezing. Depression was diagnosed based on major and recurrent episodes using the M.I.N.I.

**Results:**

*Low socioeconomic status* was associated with higher insulin resistance precursor (TG/HDL ratio) (standardized coefficient, SC = 0.064; *p* = .027) and with higher levels of depression (SC = 0.084; *p* = .001). Higher insulin resistance precursor was associated with *asthma* (SC = 0.069; *p* = .042). *Asthma* was associated with depression (SC = 0.078; *p* < .001). Lower insulin resistance precursor levels were observed in girls (SC = −0.254; *p* < .001); meanwhile, obesity (SC = 0.124; *p* < .001) and depression (SC = 0.165; *p* < .001) had higher values for them.

**Conclusion:**

Our findings underscore interconnected issues of low socioeconomic status, metabolic risk, asthma, and depression in adolescents. Recognizing the syndemic framework can lead to more effective health policies to prevent long‐term physical and mental health consequences.

**Key message**

These findings emphasize the need for integrated health interventions targeting both metabolic and mental health issues in adolescents, particularly among socially vulnerable groups. Addressing socioeconomic disparities could help reduce the burden of insulin resistance, asthma, and depression.

## 1 INTRODUCTION

Adolescence is a critical period of human development; during this stage, exposure to environmental stressors can lead to the onset of non‐communicable diseases (NCDs), which can significantly exacerbate their long‐term effects impact.^1^ Asthma is one of the most prevalent NCDs in young people, resulting from a complex interaction between genetic predisposition, environmental triggers, and behavioral factors, and it contributes significantly to the global burden of disease.^2^ Asthma, beyond its direct effects on respiration, is increasingly recognized as part of a broader range of interconnected health issues that often begin in early life.^3^, ^4^

At the same time, depression has been acknowledged as a mental NCD due to its chronic course and substantial global impact. Rather than occurring in isolation, depression frequently coexists with physical illnesses, sharing common risk factors and social determinants. A growing piece of evidence shows that adolescents with asthma are at higher risk for developing depression.^5^, ^6^, ^7^, ^8^ This relationship appears to be bidirectional, with each condition potentially worsening the other.^9^, ^10^, ^11^ Proposed mechanisms include elevated systemic inflammation and disturbances in the hypothalamic–pituitary–adrenal (HPA) axis, which can disrupt cortisol balance and contribute to both increased airway inflammation and greater asthma severity.^9^, ^10^, ^11^

Metabolic risk factors, especially insulin resistance and obesity, may be beyond the complex relationship between asthma and depression. Insulin resistance, often driven by systemic inflammation, may increase airway hyperreactivity and worsen asthma symptoms.^12^, ^13^ In addition, obesity, a frequent underlying factor of insulin resistance, contributes to this inflammatory state by releasing cytokines and adipokines from excess fat tissue, which can further impair respiratory function.^14^ Related to depression, a meta‐analysis of 70 studies found increased insulin resistance in depressed adults, with no significant changes following antidepressant treatment.^7^ In youth with obesity, higher levels of insulin resistance were associated with increased neuroimaging findings as reduced volumes in the hippocampus and anterior cingulate cortex (ACC), accompanied by more severe depressive symptoms.^15^ Additionally, there is consistent evidence of a bidirectional relationship between obesity and depression.^16^

Thus, asthma and depression often co‐occur in adolescence, potentially driven by shared metabolic risk factors, such as hyperglycemia and dyslipidemia, which are core features of insulin resistance and obesity. These conditions tend to cluster, primarily driven by socioeconomic disparities, where their interaction creates a syndemic framework. Syndemic refers to the synergistic interaction of two or more health conditions that share common risk factors, with their effects intensified by adverse social determinants.^17^

Although previous studies have explored the links between these conditions, often examining them in pairs, none have examined the syndemic interaction of insulin resistance, obesity, asthma, and depression in adolescents while accounting for social determinants. This study aims to fill that gap by proposing a comprehensive syndemic model that integrates metabolic and social determinants to elucidate the clustering of insulin resistance, asthma, and depression in adolescents, offering a deeper understanding of these interconnected conditions within the context of social vulnerability.

## 2 METHODS

### 2.1 Study design

This cross‐sectional, population‐based study was nested within the RPS Birth Cohort Consortium in São Luís, Brazil. The Federal University of Maranhão Ethics Committee (IRB #1.302.489) approved the study, and written informed consent was obtained from all participants and their legal guardians. Data were obtained from adolescents born in São Luís who participated in the second follow‐up conducted between January and November 2016. During this period, we located 687 individuals from the original prospective cohort. To increase the sample size and ensure representativeness of adolescents born in 1997–1998 living in São Luís at the time of follow‐up, we additionally recruited new participants from the same birth years using random selection from the Brazilian Live Birth Information System (SINASC) database (*n* = 1133). Further eligible adolescents were identified in schools and universities, provided they were born in São Luís in 1997–1998 (*n* = 695). Thus, the final sample comprised 2515 participants, combining individuals from the original prospective cohort with those retrospectively recruited to strengthen population representativity.

### 2.2 Data collection procedures

The data was collected by trained interviewers using a structured questionnaire to elicit socioeconomic information, including sex (dichotomized into 1‐ male and 2‐ female); educational level of the head of the family and the adolescent (college, high school, elementary school and no formal education); economic class [categorized as A/B, C, and D/E based on the Brazilian Economic Classification (CEB in the Portuguese acronym) criterion, with D/E being the lowest economic class and A, the highest]; monthly household income in 2016 (equivalent to US$270.76), categorized into (1) ≥5; (2) <5 to ≥3; (3) <3 to ≥1; and (4) <1. *Low Socioeconomic Status* was modeled as a latent variable inferred from the shared variance among its indicators: educational level of the head of the family and the adolescent, economic class, and monthly household income.

The diagnosis of depression was obtained by applying the Mini *International Neuropsychiatric Interview* – *Brazilian version 5*.0 – DSM IV (M.I.N.I.). The M.I.N.I. is a standardized diagnostic interview compatible with the diagnostic criteria of the DSM‐IV (Diagnostic and Statistical Manual of Mental Disorders – Fourth Edition). The diagnosis of depression was based on the presence of major depressive episodes and recurrent major depressive episodes.^18^

Asthma symptoms were evaluated using a structured questionnaire. *Asthma* was modeled as a latent variable, inferred from the shared variance of four categorical indicators: history of wheezing (“Have you ever had wheezing?”; yes/no), history of asthma (“Have you ever had asthma?”; yes/no), physician‐diagnosed asthma or bronchitis (“Has a doctor ever told you that you had asthma or bronchitis?”; yes/no), and current wheezing (“Do you still have wheezing?”; yes/no). These indicators collectively captured lifetime and current respiratory symptoms, allowing asthma to be represented as an underlying construct rather than a single dichotomous measure.

Blood was taken for biochemical markers, including triglyceride (TG) levels (mg/dL) and HDL levels (mg/dL). The ratio of triglycerides and HDL‐cholesterol (TG/HDL‐c) was used as a metabolic marker of insulin resistance precursor.^19^ The TG/HDL ratio was analyzed in tertiles to reflect increasing levels of metabolic impairment.

Standard techniques were used to measure the adolescents' weight and height. Weight and body composition measurements were taken using a Body Scan Cosmed body composition gauge and an Alturexata anthropometer. Obesity was classified according to World Health Organization standards using the z‐score of body mass index [BMI (kg/m^2^)] for age and sex using the following cut‐offs: non‐obese (≤ + 2 SD), and obese (> + 2 SD).^20^

### 2.3 Proposed theoretical model

**FIGURE 1:**
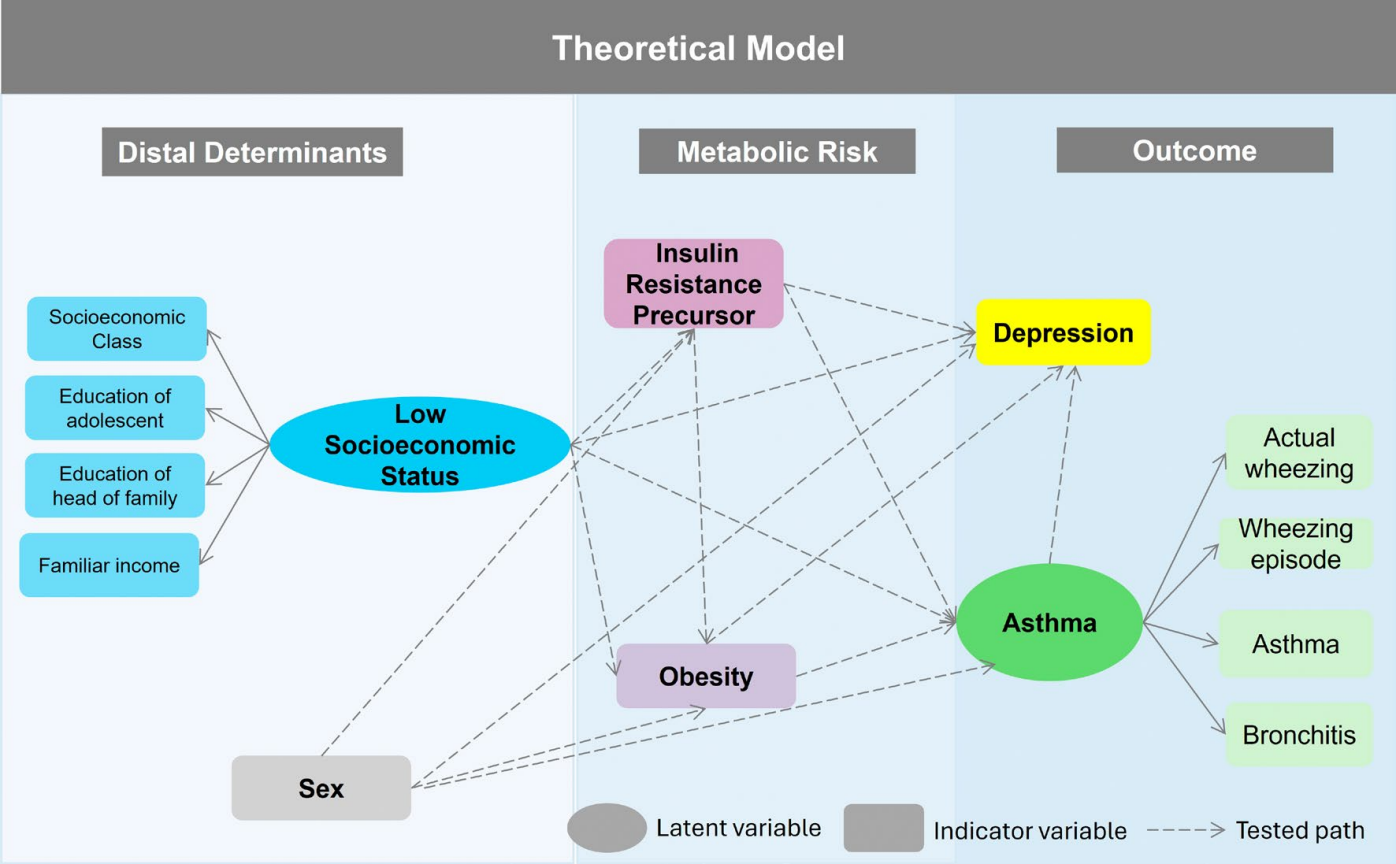
Theoretical model proposed to analyze a syndemic framework between insulin resistance, asthma, and adolescent depression. São Luís, Brazil (2023).

To analyze the syndemic framework between social vulnerability, insulin resistance, asthma, and depression, we depict a theoretical model (Figure 1). In this theoretical model, latent variables are represented by circles. The latent variable *Low Socioeconomic status* was considered a distal determinant of the model and influenced the other variables. The insulin resistance (TG/HDL ratio) and obesity were intermediate variables that marked metabolic risk, and depression and *asthma* were the outcomes of interest. The selection of variables for the theoretical model was guided by the study's objective of evaluating socioeconomic, metabolic, and psychosocial interrelations within a syndemic framework. Insulin resistance precursor (TG/HDL‐c ratio), obesity, and depression were included as core indicators of metabolic and psychological vulnerability due to their established relevance in adolescent health and their consistent availability in the RPS Consortium.

### 2.4 Statistical analysis

Categorical variables were described by absolute and relative frequencies, using STATA® statistical software, version 16.0.

The proposed theoretical model was analyzed using Structural Equation Modeling (SEM) in the Mplus version 8.0 software (Muthén & Muthén, LA, USA). SEM is a multivariable data analysis tool that models complex relations among a set of variables, allowing effect decomposition and explicitly identifying direct and mediated relationships.^21^ SEM allows us to estimate latent variables deduced from the correlation among indicator variables, representing a shared variance of phenomena, to reduce measurement errors.^22^ Convergent validity refers to the extent to which a set of observed indicators consistently measures the same underlying construct. In this context, latent variable indicators are expected to present factor loadings ideally greater than 0.50 and statistical significance (*p* < .05).^23^ Such loadings indicate that the indicators meaningfully converge to represent the same theoretical construct, supporting reliable measurement within the SEM framework.

After carrying out confirmatory factor analysis, we performed a pathway analysis. The Weighted Least Squares Mean and Variance Adjusted estimator (WLSMV) was used for the continuous and categorical variables and for multiple imputations in missing data.^22^ The THETA parameterization was used to control differences in residual variances, and the STUDYX was used to get standardized coefficients based on the respective standard deviation. *Root Mean Square Error of Approximation* (RMSEA) with an upper limit of the 90% confidence interval of less than 0.08, and CFI (*Comparative Fit Index*) and TLI (*Tucker‐Lewis Index*) >0.95 were adopted to assess the Goodness of Fit for both Confirmatory Factor and Pathway Analysis. RMSEA values below 0.05 indicate a close fit between the hypothesized model and the observed data. CFI and TLI values approaching 1.0 indicate that the proposed model performs well relative to a baseline model with no specified relationships among variables. These criteria were used to determine whether the final model provided an acceptable and theoretically coherent representation of the data.

We estimated three effects: (i) direct effects, which represent the influence of a predictor on an outcome through a single, non‐mediated pathway; (ii) indirect effects, which quantify the portion of the association transmitted through one or more mediating variables; and (iii) total effects, defined as the sum of the direct effect and all indirect pathways linking the predictor to the outcome. In SEM analyses, standardized coefficients may assume positive or negative values, reflecting direct or inverse associations between exposures and outcomes. A positive coefficient indicates that higher levels of the predictor are associated with higher levels of the outcome, whereas a negative coefficient denotes an inverse relationship.

## 3 RESULTS

Of the 2515 adolescents included in the study, 52.45% were female. Regarding family economic class, 44.45% of the adolescents (*n* = 1118) belonged to economic class C (medium). When assessing asthma symptoms, 16.69% (*n* = 417) had ever experienced wheezing, 11.32% (*n* = 283) reported having asthma at least once in their lifetime, 9.83% (*n* = 243) had been diagnosed with asthma or bronchitis by a physician, and 6.52% (*n* = 163) had experienced wheezing crises in the previous year. 11.8% (*n* = 296) of the adolescents were diagnosed with depression (Table 1).

**TABLE 1:** Sociodemographic and clinical characteristics and metabolic risk indicators of adolescents (18–19 year‐old), São Luís, Brazil (*n* = 2515).

Variables	*N*	%
Sex		
Male	1196	47.55
Female	1319	52.45
Family income, Brazilian monthly minimum wage		
≥5	440	17.50
≥3 to <5	264	10.50
≥1 to <3	1247	49.58
<1	290	11.53
Missing	274	10.89
Educational level of head of family		
College	325	12.92
Incomplete college	81	3.22
High school	1260	50.10
Elementary school	563	22.39
No formal education	286	11.37
Family economic class (ABEP)		
A	94	3.74
B	565	22.47
C	1118	44.45
D/E	450	17.89
Missing	288	11.45
Have you ever had wheezing in your chest?		
No	2082	81.31
Yes	417	16.69
Have you ever had asthma?		
No	2216	88.68
Yes	283	11.32
Has your doctor ever told you that you had asthma or bronchitis?		
No	2228	90.17
Yes	243	9.83
Wheezing crisis		
No	2336	93.48
Yes	163	6.52
Obesity		
Eutrophic	1905	75.75
Overweight	459	18.25
Obesity	151	6.00
Depression		
No	2219	88.20
Yes	296	11.80
Insulin resistance precursor (TG/HDL‐ratio)		
1st tertile	774	30.97
2nd tertile	866	34.65
3rd tertile	859	34.37

The parameters of Goodness‐of‐fit (RMSEA = 0.046; 90%CI: 0.041–0.052; CFI = 0.94; TLI = 0.90) were considered excellent (Table 2). The effect indicators of the latent variables, *Low Socioeconomic Status* and *Asthma*, showed good convergent validity with a *p*‐value <.001 (Table 3).

**TABLE 2:** Measures of fit of the structural equation model.

Model fit indices	Expected indices	Model Indexes
_*X*_^2^ ^a^		95.983
Degrees of freedom		53
*p* value *X* ^2^		0.0000
RMSEA^b^	<0.05	0.046
90% CI^c^	<0.08	0.041–0.052
*p* ^d^	>0.05	0.87
CFI^e^	>0.90	0.94
TLI^f^	>0.90	0.90

^a^ Chi‐squared test.

^b^ Root mean square error of approximation.

^c^ Confidence interval.

^d^ *p* value.

^e^ Comparative fit index.

^f^ Tucker Lewis index.

**TABLE 3:** Factor load, standard error, and *p*‐value for the latent variable framework effect indicators.

Latent variables	Standardized coefficients	Standard error	*p*
Low Socioeconomic Status			
Family income	0.601	0.029	<.001
Mother's schooling	0.563	0.025	<.001
Adolescent schooling	0.666	0.030	<.001
Economic class	0.636	0.035	<.001
Asthma			
Have you ever had wheezing in your chest?	0.394	0.023	<.001
Have you ever had asthma?	0.731	0.023	<.001
Has your doctor ever told you that you had asthma or bronchitis?	0.901	0.025	<.001
Wheezing Crisis	0.253	0.023	<.001

According to the model analysis, *Low Socioeconomic Status* was directly associated with higher tertiles of the insulin resistance precursor (SC = 0.064; *p* = .027) and with higher levels of depression (SC = 0.084; *p* = .001), indicating that adolescents from lower socioeconomic backgrounds tended to show greater metabolic and psychological vulnerability. *Low Socioeconomic Status* was also inversely associated with *Asthma* (SC = −0.080; *p* = .036).

Higher levels of the insulin resistance precursor were directly associated with obesity (SC = 0.197; *p* < .001) and positively associated with *asthma* (SC = 0.069; *p* = .042), highlighting the role of metabolic dysregulation in these outcomes.

*Asthma* was positively associated with depression (SC = 0.078; *p* < .001), suggesting an interplay between respiratory and psychological health in adolescence.

**FIGURE 2:**
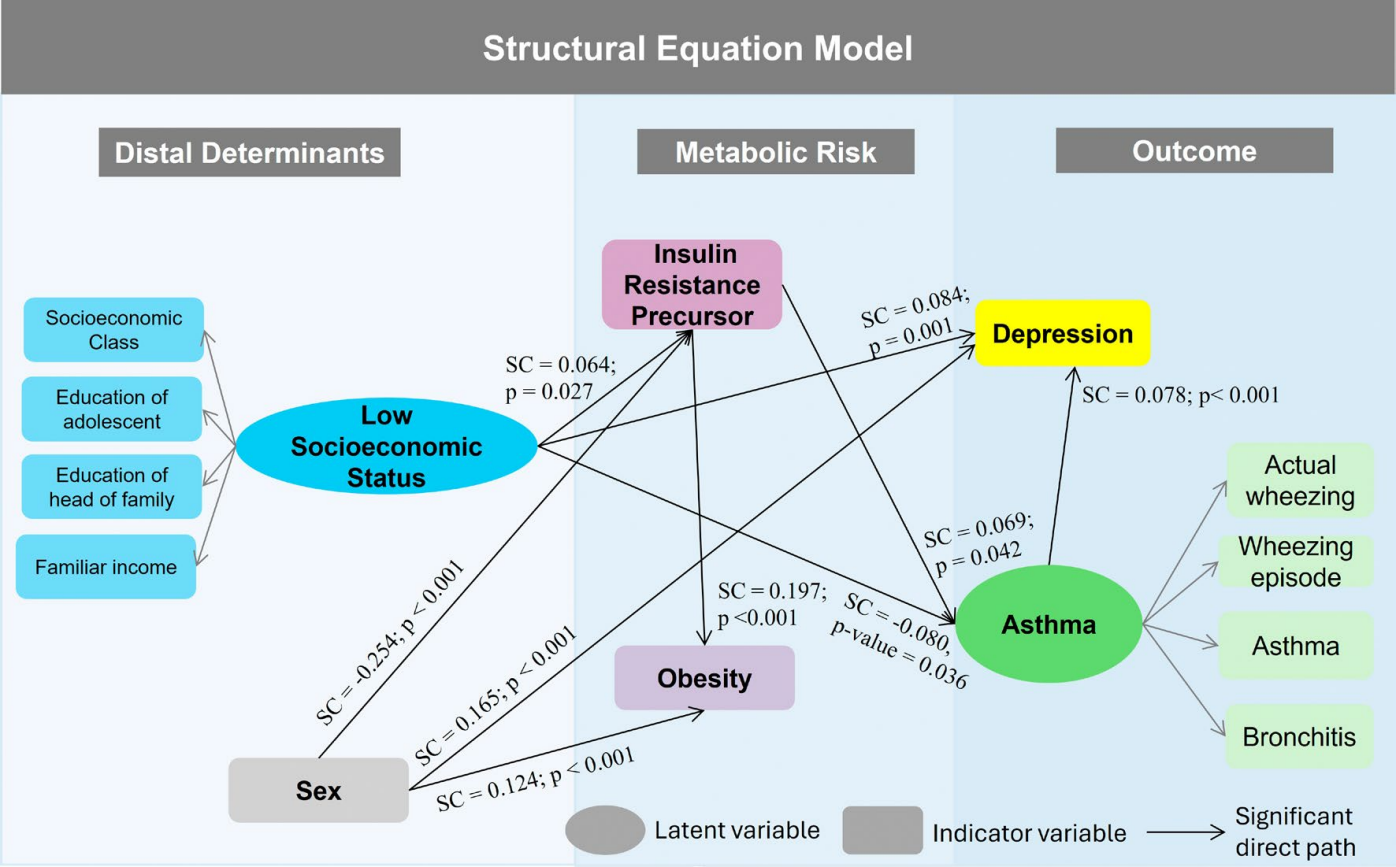
Structural Equation Model illustrating the associations between low socioeconomic status, insulin resistance, asthma, and depression. Only significant direct pathways are shown. *SC: Standardized coefficient.

Female adolescents showed higher levels of obesity (SC = 0.124; *p* < .001) and depression (SC = 0.165; *p* < .001), but lower levels of an insulin resistance precursor (SC = −0.254; *p* < .001) than males (Figure 2).

All direct and indirect effects estimated by SEM are available in Tables S1–S3.

## 4 DISCUSSION

Our findings provide empirical support for a syndemic model in adolescence, revealing that obesity and insulin resistance are interconnected, and that insulin resistance is significantly associated with *Asthma*. In turn, *Asthma* showed a direct association with depression, illustrating a chain of interactions among physical and mental health conditions. Moreover, the interplay of these conditions was amplified by social vulnerability, as *Low Socioeconomic Status* was associated with higher levels of insulin resistance and depression.

One limitation of this study is its cross‐sectional design, which precludes us from establishing a cause‐and‐effect relationship among the observed connections. However, our primary aim was not to demonstrate causality but instead to highlight the co‐occurrence of chronic conditions—such as metabolic risk linked to insulin resistance, asthma, and depression—particularly in the context of social inequalities. This indicates a syndemic framework affecting young people. In addition, we primarily investigated an asthma phenotype linked to metabolic risk. Therefore, other well‐known asthma risk factors—such as concurrent atopic conditions, family history of asthma, and environmental exposures (e.g., passive smoking) were not included in the present models.

A strength of our study is the use of a latent variable approach, which reduces measurement error when identifying social inequalities and asthma. Additionally, depression and obesity were accurately assessed. Depression was defined using the Mini International Neuropsychiatric Interview (M.I.N.I.), a tool with high validity and reliability in diagnosing psychiatric disorders. Obesity was evaluated using the BOD POD, a precise and gold‐standard method for body composition analysis.^24^ As a novelty, we explored the interconnected relationships of social determinants and metabolic risk factors in the grouping of asthma and depression among youth.

Insulin resistance, a precursor to metabolic syndrome, was associated with asthma in adolescents, indicating a metabolic risk factor linked to respiratory issues. Our findings indicate that the relationship between insulin resistance and asthma was independent of obesity. Hyperlipidemia showed a positive association with the prevalence of asthma in Korean adolescents, especially higher serum total cholesterol levels and TG/HDL‐c ratio as independent risk factors for asthma,^25^ as in our study.

Increased blood cholesterol may be involved in the occurrence and development of asthma.^26^, ^27^, ^28^ As an explanation, a potential mechanism for the association between dyslipidemia and asthma may be related to inflammation, as hypercholesterolemia plays a pro‐inflammatory role by inducing the release of inflammatory cytokines and positively regulating cell adhesion molecules on the endothelium, which characterizes asthma through chronic inflammation of the airways.^29^ Hypercholesterolemia alters the immune response and Toll‐like receptor signaling in macrophages. High cholesterol can reduce the mRNA expression of T‐cell‐related cytokines, altering the Th1/Th2 ratio, in which Th2 cells and their cytokines can control asthma, leading to the accumulation of eosinophils in the airway wall, excessive mucus production, and promoting the synthesis of immunoglobulin E by allergen‐specific B cells, ultimately causing an asthma attack.^30^

In our more proximal findings, *asthma* was associated with depression. These data are in line with a retrospective cohort from Korean National Health Insurance Service (NHIS) data in which patients with asthma were associated with the subsequent development of depressive disorders with a hazard ratio (HR) of 1.35 (1.31–1.40).^31^ Chronic inflammation, a hallmark of asthma, has been implicated in the pathophysiology of depression through elevated levels of pro‐inflammatory cytokines such as IL‐6 and TNF‐α, which may contribute to neuroinflammation and altered neurotransmitter function. Additionally, dysregulation of the hypothalamic–pituitary–adrenal (HPA) axis in response to chronic stress from asthma management may exacerbate depressive symptoms.^9^, ^10^, ^11^

Characterizing the social dimension of this syndemic framework, we observed that *Low Socioeconomic Status* was a determinant of metabolic risk, with an increase in the insulin resistance precursor. As an explanation, social vulnerability exposes families to food insecurity, resulting in lower consumption of fruits and vegetables and more processed foods, which, in some cases, are cheaper and more accessible.^32^ No significant association was found between *Low Socioeconomic Status* and obesity, supporting the fact that the relationship between socioeconomic disadvantage and obesity remains inconsistent across studies,^33^, ^34^ potentially influenced by the stage of nutritional transition.^35^ The global nutrition transition has increased the double burden of malnutrition (undernutrition or obesity), impacting health across the life course, disrupting the gut microbiome, metabolism, and insulin signaling, fostering chronic inflammation, and increasing the risk of non‐communicable diseases.^36^

*Low socioeconomic status* was inversely associated with *asthma* and directly associated with depression. While low socioeconomic status is a known risk factor for various noncommunicable diseases, certain aspects may protect against asthma. For instance, the hygiene hypothesis suggests that early exposure to a diverse microbial environment, often found in lower‐income settings, can strengthen the immune system and reduce the risk of asthma.^37^, ^38^ Social determinants are a well‐established risk factor for depression. There is a dose–response relationship between socioeconomic status and depression, so with each additional year of education or percentage increase in relative income, the chances of becoming depressed decrease by up to 3%, even among adolescents.^39^ As a possible explanation, it has been shown that the lasting impression of low economic status affects gene expression patterns in the blood in adulthood, so that individuals with low socioeconomic status in early life show transcriptional profiles indicating increased pro‐inflammatory signaling and decreased glucocorticoid signaling even after possibly higher socioeconomic circumstances during adulthood.^39^

In our study, young females were found to be at greater risk of obesity and depression. A meta‐analysis evaluating the bidirectional associations between depression and obesity in adolescents revealed that, while this relationship exists for both sexes, it is notably stronger for females.^16^ Other reviews further corroborate a gender‐specific effect, with females exhibiting a more pronounced association between obesity and depression.^40^, ^41^

Interestingly, our findings did not identify an association between obesity and depression in adolescents. This discrepancy may be attributed to the low prevalence of obesity in our study population (6%), which is significantly lower than that reported in other populations.^42^ However, we observed an association between another metabolic risk factor, the TG/HDL ratio, and depression, suggesting that other metabolic parameters may play a role in the relationship between metabolic health and mental health in adolescents. Additionally, male sex was associated with higher insulin resistance, which may be partly explained by greater visceral fat accumulation and differences in hormonal regulation, such as lower estrogen levels and higher androgen activity, which influence glucose metabolism and insulin sensitivity.^43^

This study emphasizes a syndemic framework that links lower socioeconomic status to metabolic risk, including insulin resistance, asthma, and depression. This insulin resistance risk is also associated with obesity and asthma in adolescents. These results highlight the syndemic nature of these conditions and the need for integrated strategies that address both behavioral and social determinants.

## AUTHOR CONTRIBUTIONS

**Aline Sousa Falcão:** Writing – original draft; visualization. **Lorena Lúcia Costa Ladeira:** Writing – review and editing; formal analysis; visualization. **Susilena Arouche Costa:** Formal analysis; writing – review and editing; visualization. **Rosângela Fernandes Lucena Batista:** Methodology; validation; writing – review and editing; visualization. **Claudia Maria Coelho Alves:** Methodology; validation; visualization; writing – review and editing. **Erika Bárbara Abreu Fonseca Thomaz:** Methodology; validation; visualization; writing – review and editing. **Cecília Claudia Costa Ribeiro:** Conceptualization; methodology; validation; visualization; writing – review and editing; formal analysis.

## FUNDING INFORMATION

This study was funded by FAPEMA (Maranhão State Foundation for Research and Scientific and Technological Development), DECIT (Department of Science and Technology), CNPq (National Council for Scientific and Technological Development), and CAPES (Coordination for the Improvement of Higher Education Personnel) Finance Code 001.

## CONFLICT OF INTEREST STATEMENT

The authors have stated explicitly that there are no conflicts of interest in connection with this article.

## Supporting information


Table S1.

## Data Availability

The data supporting this study's findings are available from the corresponding author upon reasonable request.
