# Supplementary material for: Low socioeconomic status, insulin resistance, asthma, and depression: A syndemic framework in Brazilian adolescents
Source: Pediatr Allergy Immunol. 2026 Feb 22;37(2):e70290. doi: 10.1111/pai.70290 (PMC12926274; doi:10.1111/pai.70290)
Supplement: Supplementary file 1 — Table S1. [file PAI-37-e70290-s001.docx]

| **Independent**  **Variable** | **Dependent**  **Variable** | **Standardizated**  **coefficient** | **p-value** |
| --- | --- | --- | --- |
| **SES** | **Asthma** | **-0.080** | **0.036** |
| Sex | Asthma | -0.025 | 0.413 |
| **Insulin Resistance** | **Asthma** | **0.069** | **0.042** |
| Obesity | Asthma | 0.029 | 0.472 |
| **SES** | **Insulin Resistance** | **0.064** | **0.027** |
| SES | Obesity | -0.032 | 0.375 |
| **SES** | **Depression** | **0.084** | **0.001** |
| **Asthma** | **Depression** | **0.078** | **0.004** |
| **Sex** | **Insulin Resistance** | **-0.254** | **<0.001** |
| **Sex** | **Obesity** | **0.124** | **<0.001** |
| **Insulin Resistance** | **Obesity** | **0.197** | **<0.001** |
| **Sex** | **Depression** | **0.165** | **<0.001** |
| Insulin Resistance | Depression | 0.008 | 0.754 |
| Obesity | Depression | 0.024 | 0.377 |

**Table S1.** All direct effects estimated by SEM, n =2,515.

**Table S2.** Total and total indirect pathways estimated by SEM, n=2,515.

| **Effect** | **Pathway** | **Standardizated**  **coefficient** | **p-value** |
| --- | --- | --- | --- |
| **Total** | Ses → Asthma | **-0.076** | **0.046** |
| **Total indirect** | Ses → Asthma | 0.004 | 0.269 |
| **Total** | Insulin Resistance→ Asthma | **0.075** | **0.022** |
| **Total indirect** | Insulin Resistance→ Asthma | 0.006 | 0.474 |
| **Total** | Obesity → Asthma | 0.029 | 0.472 |
| **Total indirect** | Obesity → Asthma | 0.000 | 1.000 |
| **Total** | Ses → Insulin Resistance | **0.064** | **0.027** |
| **Total indirect** | Ses → Insulin Resistance | 0.000 | 1.000 |
| **Total** | Insulin Resistance → Depression | 0.018 | 0.437 |
| **Total indirect** | Insulin Resistance → Depression | 0.011 | 0.096 |
| **Total** | Ses → Depression | **0.078** | **0.003** |
| **Total indirect** | Ses → Depression | -0.006 | 0.171 |

**Table S3.** Indirect pathways estimated by SEM, n=2,515.

| **Indirect Pathway** | **Standardizated**  **coefficient** | **p-value** |
| --- | --- | --- |
| Ses → Obesity → Asthma | -0.001 | 0.576 |
| Ses → Insulin Resistance → Asthma | 0.004 | 0.143 |
| Ses → Obesity → Insulin Resistance → Asthma | 0.000 | 0.496 |
| Insulin Resistance → Obesity → Asthma | 0.006 | 0.474 |
| Insulin Resistance → Obesity → Depression | 0.005 | 0.382 |
| Insulin Resistance → Asthma → Depression | 0.005 | 0.106 |
| Insulin Resistance → Asthma → Obesity → Depression | 0.000 | 0.491 |
| Ses → Obesity → Depression | -0.001 | 0.548 |
| Ses → Insulin Resistance → Depression | 0.000 | 0.754 |
| Ses → Asthma → Depression | -0.006 | 0.107 |
| Ses → Obesity → Insulin Resistance → Depression | 0.000 | 0.418 |
| Ses → Asthma → Obesity → Depression | 0.000 | 0.583 |
| Ses → Asthma → Insulin Resistance → Depression | 0.000 | 0.198 |
| Ses → Asthma → Obesity → Insulin Resistance → Depression | 0.000 | 0.512 |
